# Lifetime Variations of Prolactin Receptor Isoforms mRNA in the Hippocampus and Dentate Gyrus of the Rat—Effects of Aging

**DOI:** 10.3390/ijms26115023

**Published:** 2025-05-23

**Authors:** Marta Carretero-Hernández, Elisa Herráez, David Hernández-González, David Díez-Castro, Leonardo Catalano-Iniesta, Josefa García-Barrado, Enrique J. Blanco, José Carretero

**Affiliations:** 1 Department of Human Anatomy and Histology, Faculty of Medicine, University of Salamanca, 37007 Salamanca, Spain; davidhnz@usal.es (D.H.-G.); dadicas@usal.es (D.D.-C.); leonardo.catalano@usal.es (L.C.-I.); ejbb@usal.es (E.J.B.); jcar@usal.es (J.C.); 2 Laboratory of Neuroendocrinology, Institute of Neuroscience of Castilla y León, University of Salamanca, 37007 Salamanca, Spain; barrado@usal.es; 3 Laboratory of Neuroendocrinology and Obesity, Biosanitary Institute of Salamanca (IBSAL), 37007 Salamanca, Spain; 4 Department of Physiology and Pharmacology, Faculty of Medicine, University of Salamanca, 37007 Salamanca, Spain; elisah@usal.es; 5 Experimental Hepatology and Drug Targeting (HEVEPHARM) Group, Institute of Biomedical Research of Salamanca (IBSAL), University of Salamanca, 37007 Salamanca, Spain; 6 National Institute for the Study of Liver and Gastrointestinal Diseases (CIBERehd, “Instituto de Salud Carlos III”), 28029 Madrid, Spain

**Keywords:** hippocampus, short prolactin receptor, large prolactin receptor, aging

## Abstract

Prolactin is a hormone for which actions on the central nervous system such as neurogenesis and neuroprotection have been described by acting on specific receptors. The presence of prolactin receptors in the brain, including the hippocampus, is well documented; however, it is unknown whether these receptors change with age and whether they are related to sex. For this reason, a study of the expression of prolactin receptors, in the short and long isoforms, in the hippocampus of male and female rats has been carried out by qPCR and in situ hybridization, with a densitometric analysis in the following life stages: prepubertal, postpubertal, young adult, adult, and old. The results revealed the greater expression of the long isoform than of the short isoform in males, but not in females, with significant differences between males and females and in the different life stages studied. With significant differences, the highest expression of both isoforms appeared in male rats in the postpubertal stage, and the lowest expression was observed in adult and old animals. In situ hybridization showed differences in the localization of PRLR mRNA expression in CA1, CA3, and DG depending on the age and sex of the rats. The results obtained suggest that hippocampal aging is related to a decrease in prolactin receptors, which helps to better understand brain aging.

## 1. Introduction

Prolactin was described as a pituitary hormone related to lactation [1,2]. In the last 20 years, a large number of biological effects have been described for prolactin, including neurogenic, antioxidant, and neuroprotective effects on the brain [3,4,5,6,7,8,9,10,11].

The biological actions of prolactin are mediated by binding the hormone to a specific receptor, which belongs to the transmembrane type I cytokine receptor superfamily. The prolactin receptor is divided into three parts: extracellular, transmembrane, and intracellular domains. All receptor isoforms have the same structure in their extracellular and transmembrane domains. Therefore, the differences among isoforms depend on the structure of the intracellular domain. This domain is larger than the other two domains and interacts with other cytoplasmic molecules to initiate signaling pathways [12].

Prolactin ligands with two different extracellular interactive sites, called binding domains, induce conformational changes in the receptor dimer.

The intracellular domain structure can be divided into hydrophobic and interactive-binding sites. Some hydrophobic parts can interact with lipids and their structure changes and become capable of interacting with the inner side of the cell membrane after prolactin ligands. The interactive sites are called Box1 and Box2 [13]. The interactive sites are responsible for the existence of three isoforms: long, intermediate, and short [14].

The long isoform of the receptor is the dimerization of two receptors that contain both Box1 and Box2. The intermediate isoform is the result of the dimerization of one molecule with Box1 and Box2, but the other molecule has only Box1. The short isoform is the dimerization of two molecules that contain only Box1 [15].

Box1 activates JAK2 located near the cytoplasm via phosphorylation. This is the main difference between prolactin activity and other cytokines because JAK2 is not part of the receptor molecule. JAK2 activation initiates a signaling pathway that can be divided into three pathways: MAPK, STAT5, and Akt [15].

The activation of JAK2 provokes a series of interactions and cascade reactions that would be, in a very simplified way, Src phosphorylation, which leads to the activation of Ras, Raf, MEK, and MAPKs, ultimately stimulating cell proliferation. Another consequence of Src activation is the reaction cascade of PI3K, Akt phosphorylation, and mTor, which leads to anti-apoptotic effects. The latter pathway is also initiated by the phosphorylation of JAK2, which facilitates the phosphorylation of the STAT family. The most relevant are STAT5 and STAT3, which are internalized into the nucleus and activate their transcriptomic functions, which are typical of cytokines and growth factors [16].

Since the seventies, the presence of prolactin receptors in the brain [17], particularly in the hippocampal Ammon’s horn and dentate gyrus [18,19,20,21,22], has been known.

However, no studies in the scientific literature have analyzed possible variations in the prolactin receptor in the hippocampus during aging. It is unknown whether the neurodegeneration and memory loss that occur during late age are accompanied by significant variations in the prolactin receptor hippocampal levels.

Therefore, we analyzed whether the prolactin receptor changes during different stages of life. To determine this, we designed an experimental study in which we analyzed the expression and location of prolactin receptor isoforms in the rat hippocampus (Ammon’s horn and Dentate Gyrus) to determine if there are aging-related variations or age-related sexual dimorphism, and if this expression is related to the puberty and fertility of the animals.

## 2. Results

### 2.1. qPCR Study of Box1 and Box2

**Figure 1:**
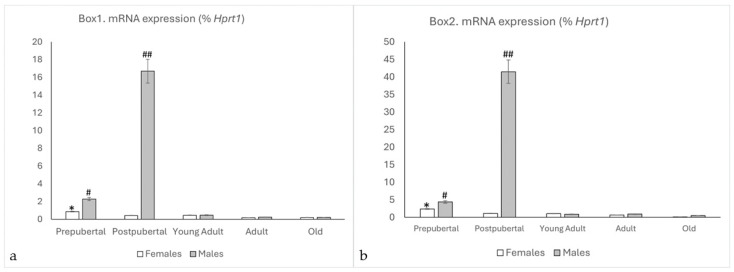
qPCR results for the mRNA of Box1 (**a**) and Box2 (**b**) in the hippocampi of female and male rats at different life stages. In the earliest stages, expression in both cases were higher in males than in females, with the expression in postpubertal males standing out. At the young adult stage, there was a clear decrease in the mRNA expression of PRLR, which was accentuated as the animals aged, highlighting the low expression of Box2 in old female rats. *: *p* < 0.01, relation with other groups of female rats. #: *p* < 0.01, relation to young adult, adult, and old males and prepubertal females. ##: *p* < 0.001 compared with the other groups of animals studied.

Prolactin receptor mRNA was detected in both sexes and age groups. Although the expression was relatively low in most of them, differential expression among males and females was observed (Figure 1a,b).

In both Box1 and Box2, female rats showed the highest expression in the prepubertal stage, which gradually decreased with age. There were significant differences in the expression of Box1 among the groups studied; high values were found in prepubertal females (*p* < 0.01, in relation to the other ages in females). Similarly, the expression of Box2 in prepubertal females showed significant differences (*p* < 0.01) compared to the rest of the studied female groups. No significant differences were observed between the remaining female groups.

For Box1 and Box2, in males, significant differences were observed when comparing postpubertal males with the other study groups (*p* < 0.001). Prepubertal males showed higher values than young adults, adults, and old males (*p* < 0.01). There were no significant differences between adult and old animals.

When comparing males and females, significant differences in Box1 and Box2 expression were observed in the prepubertal age groups, with higher expression in males than in females (*p* < 0.01), and a very significant (*p* < 0.001) peak was observed in male animals (*p* < 0.001, in relation to postpubertal females). No significant differences were observed between young, adult, and old animals.

Interestingly, both isoforms of the receptor behaved in the same way, but the expression of Box2 was at least twice that of Box1 for all groups (*p* < 0.01) in male rats, while in females, the difference was not significant.

### 2.2. In Situ Hybridization: Box1 mRNA

**Figure 2:**
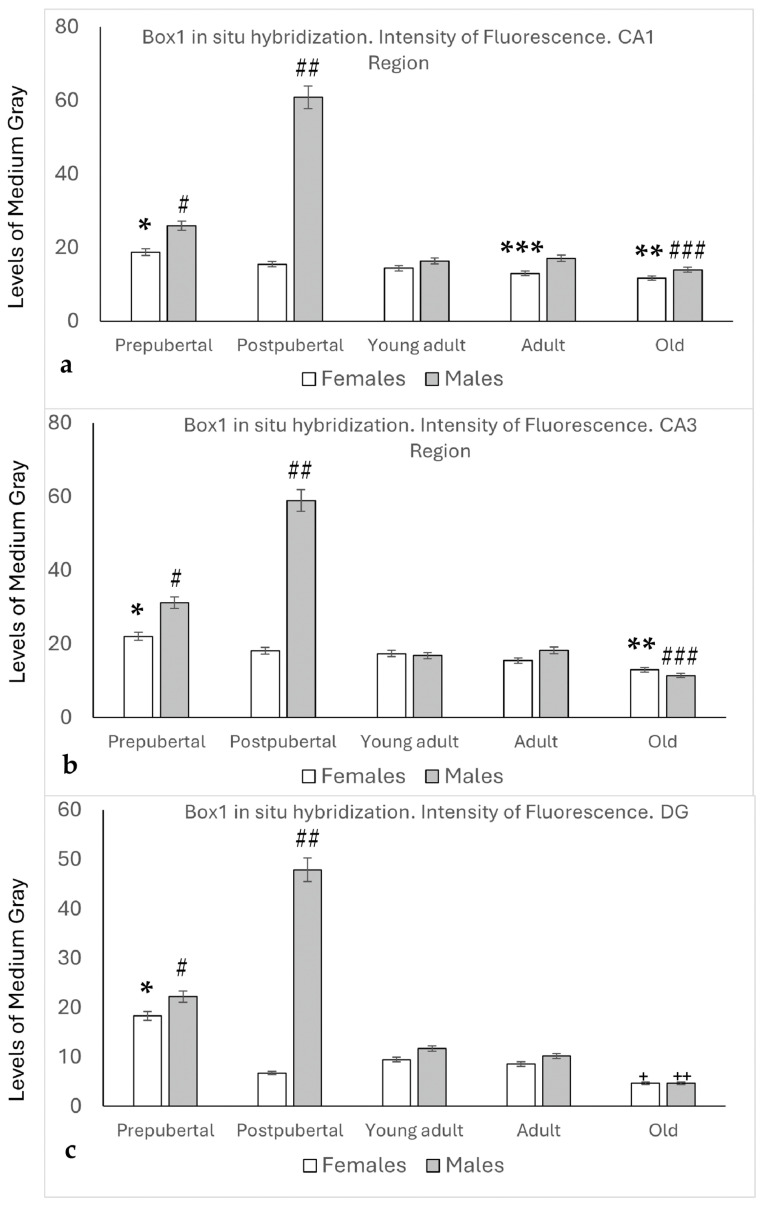
In all the regions analyzed ((**a**) CA1, (**b**) CA3, (**c**) DG), densitometric analysis of the fluorescence intensity for in situ hybridization (0: black, 255: white) showed that the highest intensity of Box1 expression occurred in postpubertal males and somewhat less in prepubertal males. Among the females, prepubertal females had the highest intensity. In both sexes, the lowest intensity was observed in the old animals. *: *p* < 0.01, relation with the other female groups. **: *p* < 0.05 in relation to postpubertal, young adult, and adult females. ***: *p* < 0.05 in relation to adult males. #: *p* < 0.05 in relation to prepubertal females, and *p* < 0.01 in relation to young adult, adult, and old males. ##: *p* < 0.001 compared to the other groups of male and female animals. ###: *p* < 0.05, compared to young adult and adult males. +: *p* < 0.05, in relation to postpubertal females and *p* < 0.01 in relation to young adults and adult females. ++: *p* < 0.01, relation to young adults and adult males.

The densitometric analysis (Figure 2) revealed that Box1 expression in the CA1 region of the Amon’s horn (Figure 2a) was significantly higher in prepubertal females than in the rest of the female groups (*p* < 0.01), and the intensity of the reaction in old females was lower than that in postpubertal, young adult, and adult females (*p* < 0.05). In prepubertal males, the intensity was higher than that in females (*p* < 0.05) or in young adults, adults, or old males (*p* < 0.01). The highest intensity values were found in postpubertal males (*p* < 0.001, compared to the other groups studied). In the CA1 region, the values observed were always higher in males than in females, although the differences were statistically significant only in prepubertal (*p* < 0.05), postpubertal (*p* < 0.001), and adult animals (*p* < 0.05).

In the CA3 region of female animals, the expression of Box1 decreased with age, with the highest intensity values found in the prepubertal stage, followed by a decrease in the postpubertal stage (*p* < 0.05) in young adult and adult females, and a significant decrease was observed in old females (*p* < 0.05) compared to postpubertal, young adult, and adult females. Significantly higher values were found in prepubertal males than in females at the same stage (*p* < 0.05). Similar to CA1, the highest intensity values were found in postpubertal males (*p* < 0.001, compared to the other groups studied). No significant differences were observed between young adults and adult males. In old males, the intensity values decreased significantly in relation to young adult and adult males (*p* < 0.05) (see Figure 2b).

The densitometric analysis of Box1 mRNA in the Dentate Gyrus (Figure 2c) showed that, except in old animals, the intensity values were higher in males than in females.

Prepubertal females showed the highest values for this sex, with significant differences (*p* < 0.01, compared to other female groups). A significant decrease was observed in postpubertal females, which partially recovered in young adult females. Values that were maintained in adult females decreased significantly in old females, which showed the lowest intensity compared to the rest of the animals and regions studied (*p* < 0.05, in relation to postpubertal females; *p* < 0.01, in relation to young adult and adult females).

In male animals, the densitometric analysis patterns were like those observed in the CA1 and CA3 regions. Higher values were observed in prepubertal males than in females (*p* < 0.05) or in young adults and adult males (*p* < 0.01). The highest values were observed in postpubertal males (*p* < 0.001, in relation to the other groups of males or females studied). Old males showed the lowest values among male animals (*p* < 0.01 in relation to young adult and adult males).

### 2.3. Distribution of Box1 mRNA in the CA1 Region by In Situ Hybridization (Figure 3)

In all groups of females studied, except adult females, the mRNA of Box1 was mainly located in the soma of neurons of the stratum pyramidal of the CA1 region, which demonstrated that the pyramidal neurons of prepubertal females were more reactive than in the other stages analyzed. Young adult and old females had positive dendrites in the radiate stratum. Adult females reacted mainly to the radiated stratum. Moreover, prepubertal females show a reaction in the dendrites of the radiate stratum close to the lacunous stratum, as can be observed in the densitometric gradient.

The largest difference between these two age groups can be seen in the densitometric gradient: in prepubertal males, the dendrites of the radiated layer were more positive than in the postpubertal males, and in these males, the pyramidal neurons were more positive than in the prepubertal males. Young adult males had some intensely positive pyramidal neurons, although with a punctiform reaction, and positivity extended to areas adjacent to the pyramidal layer of the oriens and radiated layers. In adult males, the reaction predominated in a punctiform form in the neurons of the pyramidal layer. Old males had intensely reactive pyramidal neurons and well-reactive oriens layer fibers.

**Figure 3:**
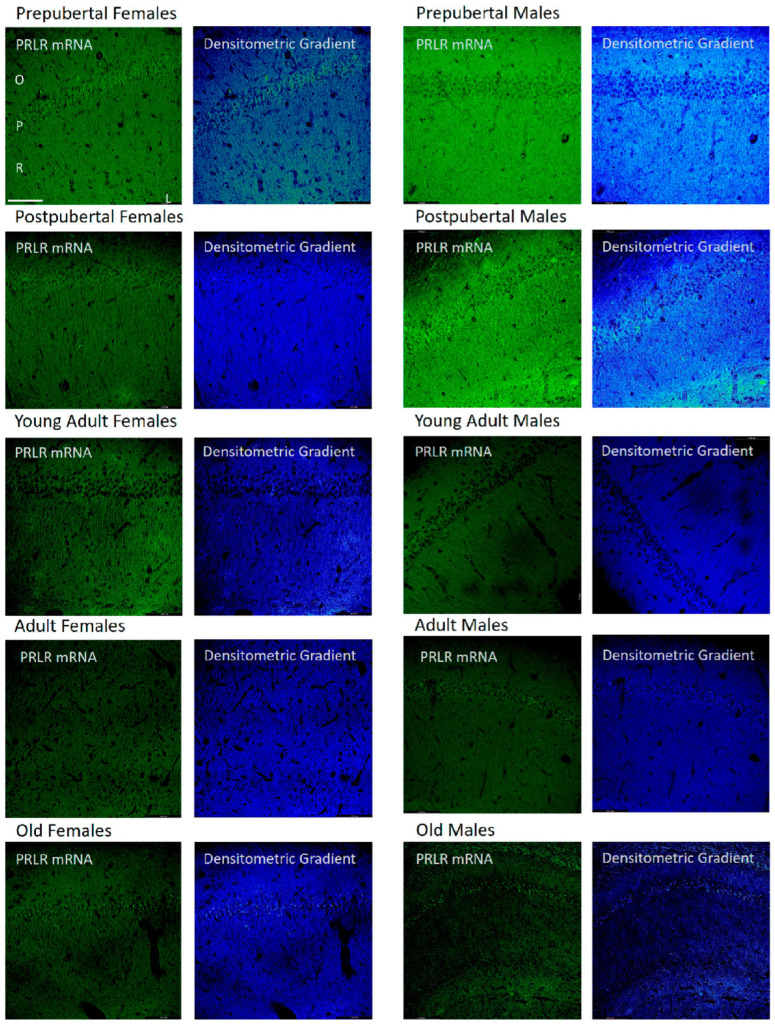
In situ hybridization showed that in CA1, except in adult female rats, the mRNA of Box1 was always located in the neurons of the pyramidal layer. Prepubertal males and, with greater intensity, postpubertal males also had reaction in the oriens and radiated layers. The lacunous layer reacted in postpubertal and old males and in postpubertal females and young adults. (O: Oriens layer, P: Pyramidal layer, R: Radiate layer, L: Lacunous Layer.) Scale bar: 116 μm.

### 2.4. Distribution of Box1 mRNA in the CA3 Region by In Situ Hybridization (Figure 4)

Patterns similar to those described for CA1 were observed in the CA3 region of pre- and postpubertal females. However, young adult females only exhibited a punctiform reaction in neurons isolated from the pyramidal layer. Adult females only showed weak positivity in some isolated pyramidal neurons, and old females showed a reaction like that of young adults, although with a higher number of positive pyramidal neurons. There were no significant differences in the distribution of positivity and its intensity with respect to those described for the CA1 region in the different groups of males studied. However, the intense positivity of some pyramidal and secondary neurons of the oriens and radiated layers in postpubertal males stands out.

**Figure 4:**
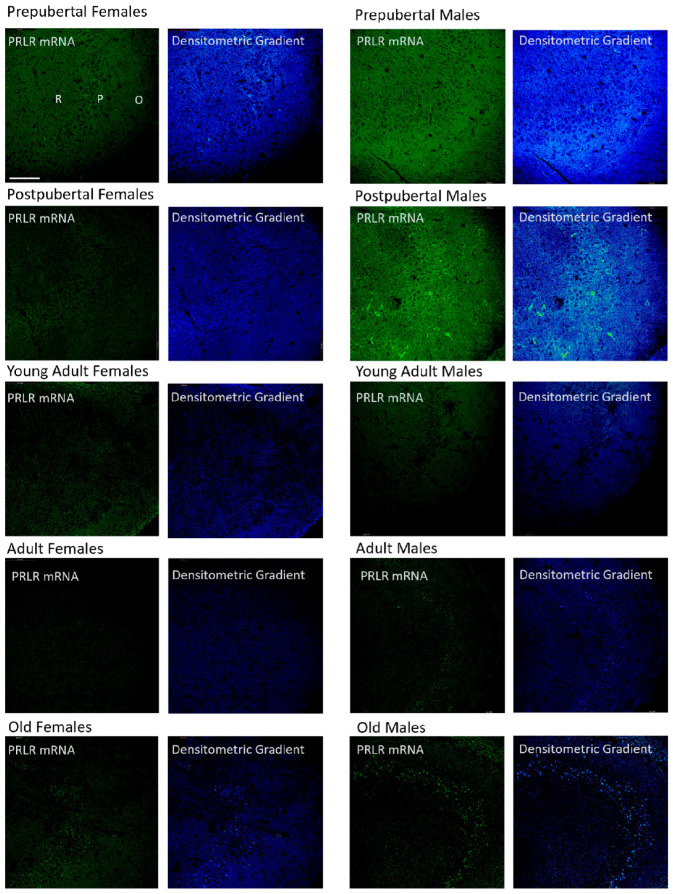
In situ hybridization showed that in CA3, the mRNA of Box1 mRNA was always located in the neurons of the pyramidal layer. In postpubertal males, the presence of neurons expressing Box1 mRNA in the oriens layer of CA3 and, to a lesser extent, in the radiated layer was striking and was not observed in the other groups of animals studied. (O: Oriens layer, P: Pyramidal layer, R: Radiate layer.) Scale bar: 116 μm.

### 2.5. Distribution of Box1 mRNA in the Dentate as Determined by In Situ Hybridization (Figure 5)

The dentate gyrus of prepubertal females showed greater positivity than that of the other groups, affecting the three layers, but was more intense in the granular and molecular layers. Postpubertal females showed positive granular neurons and the greatest reaction appeared in the marginal zone of the molecular layer. Young adult and adult females had weakly positive granular neurons along with many non-positive neurons, with the reaction predominating in the molecular and polymorphous layers. Old females had a reaction similar to that of the postpubertal females. In prepubertal and postpubertal males, an intense reaction affecting the entire dentate gyrus was observed. All granule cells and many secondary neurons were positive. Positivity was also found in all three layers of the dentate gyrus of young adult males, although at a lower intensity. Adult males had positive and negative cells, and the highest positivity was observed in the marginal area of the molecular layer. Old males showed strong positivity in the granular layer and part of the polymorphous layer. Subgranular-positive cells were observed mainly in the postpubertal males. Moreover, prepubertal males and females showed some positive cells.

**Figure 5:**
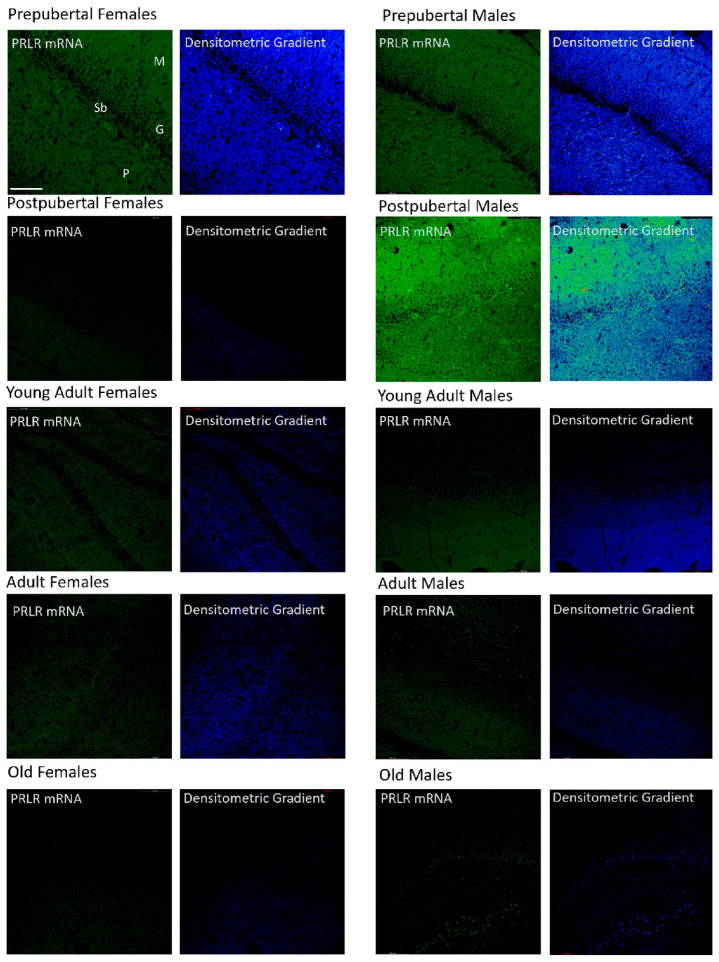
In situ hybridization showed that, in prepubertal animals and postpubertal males, Box1 mRNA was expressed in the granular cells. In postpubertal males, the expression was strong in the molecular layer and, to a lesser extent, in the polymorphic layer. From young adults to old age, the expression of Box1 in the dentate gyrus was almost non-existent. In postpubertal males and, to a lesser extent, in prepubertal males and females, subgranular cells positive for Box1 mRNA were observed. (M: Molecular layer, G: Granular layer, Sb: Subgranular layer, P: Polymorphic layer.) Scale bar: 116 μm.

### 2.6. In Situ Hybridization Study: Box2 mRNA

**Figure 6:**
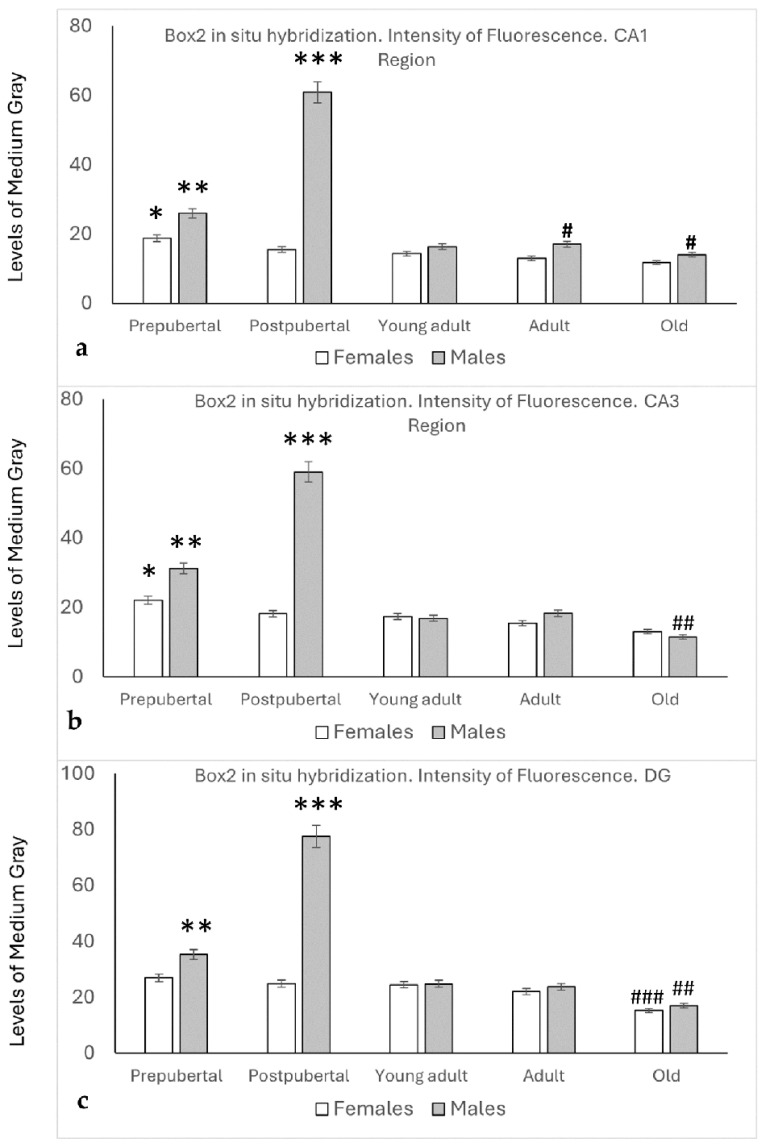
In all the regions analyzed ((**a**) CA1, (**b**) CA3, (**c**) DG), densitometric analysis of the fluorescence intensity for in situ hybridization (0: black, 255: white) showed that the highest intensity of Box2 expression occurred in postpubertal males and somewhat less in prepubertal males. Prepubertal females had the highest intensity in Ammon’s horn, but not in the dentate gyrus. In both sexes, the lowest intensity was observed in the old animals. *: *p* < 0.05, compared to adult and older females. **: *p* < 0.05 in relation to prepubertal females, and *p* < 0.01 in relation to young adult, adult, and old males. ***: *p* < 0.001 in relation to the other groups of animals studied. #: *p* < 0.05 compared to females of the same age. ##: *p* < 0.05 in relation to young adult and adult males. ###: *p* < 0.01 in relation to the other groups of female rats.

As was the case with Box1 mRNA, the presence of Box2 mRNA was detected by in situ hybridization in all the regions and groups studied. In addition, the densitometric patterns of the reaction (levels of medium gray) observed by in situ hybridization were very similar for the CA1, CA3, and the Dentate Gyrus regions (Figure 6).

The densitometric analysis of the CA1 region (Figure 6a) in female rats showed a slight decrease from the prepubertal to old stages, but only a significant difference between prepubertal, adult, and old female rats was found (*p* < 0.05).

Prepubertal males showed significantly higher values of medium gray than prepubertal females (*p* < 0.05) and young adults, adults, and old males (*p* < 0.01).

Similar to Box1 mRNA, the highest intensity was found in postpubertal males (*p* < 0.001 compared to the other groups studied). Although a slight decrease was observed in old males compared with young adults and adult males, no significant differences were found. Adult and old males showed higher values than females of the same age (*p* < 0.05).

Similar findings to those described for CA1 were found in the CA3 region, except that the values found in old males were lower than those of old females, and that the differences among young adult and adult males and old males and females were significantly lower (*p* < 0.05).

The densitometric levels of intensity in the Dentate Gyrus of female rats were similar for prepubertal, postpubertal, young adult, and adult rats. In old female rats, a significant decrease was observed compared to the other stages of age (*p* < 0.01).

The Dentate Gyrus of males showed densitometric patterns similar to those observed in the CA1 and Ca3 regions. No significant differences were observed between male and female young adult and old animals.

### 2.7. Distribution of Box2 mRNA in the CA1 Region by In Situ Hybridization (Figure 7)

In both sexes and at all age stages analyzed, an in situ hybridization positive signal for Box2 mRNA (long isoform of the prolactin receptor) can be found. Prepubertal females showed a positive reaction in the three layers analyzed, but in an irregular shape, with highly reactive neurons and fibers. Next to them, areas in which the reaction disappeared were found. In postpubertal females, the reaction increased and was homogeneously distributed, affecting all pyramidal neurons and fibers of the oriens and radiated layers, showing that the area of the greatest reaction was the radiated layer, especially the area adjacent to the lacunous stratum. In contrast, the CA1 region of young adult females showed virtually no positive reaction in any of the three layers. The positive signal in adult females was also low, but more evident than in young adult females and less evident than in old females. The reaction in the adult and old females was homogeneous across the three studied layers. Comparing the presence of Box2 mRNA between males and females, the reaction in prepubertal males was homogeneous, very similar in postpubertal males, and decreased in young adults, although some pyramidal neurons appeared to be intensely reactive in males. In adult males, the reaction increased, and unlike in females, the presence of positive pyramidal neurons was evident. In addition, unlike what was observed in females, old males showed a decrease in the reaction, even less than that observed in young adult males. Although there was some positivity in the oriens and radiated layers, the reaction was more evident and homogeneous in the pyramidal layer.

**Figure 7:**
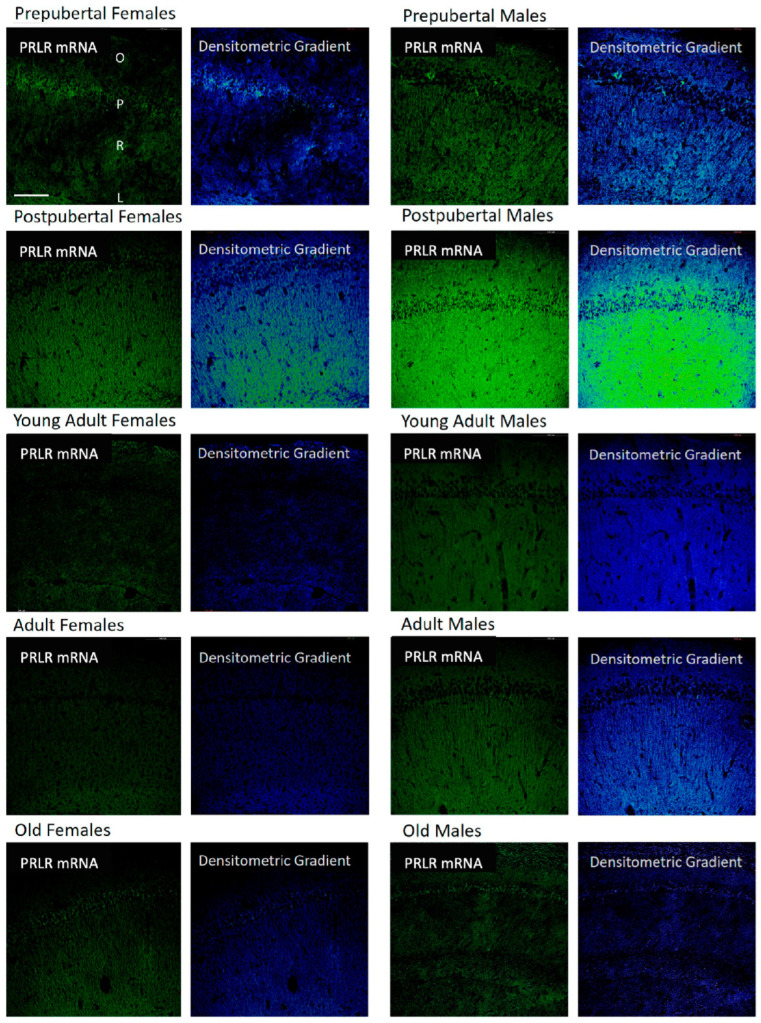
In situ hybridization showed that in CA1, except in young adult females and in most neurons of adult females, the mRNA of Box2 was always located in the neurons of the pyramidal layer. Prepubertal and, with greater intensity, postpubertal animals also had reaction in the oriens and radiated layers. The lacunous layer reacted in prepubertal, postpubertal, and old males and in prepubertal, postpubertal, and young adult females. (O: Oriens layer, P: Pyramidal layer, R: Radiate layer, L: Lacunous Layer.) Scale bar: 116 μm.

### 2.8. Distribution of Box2 mRNA in the CA3 Region by In Situ Hybridization (Figure 8)

The variations in the results of Box2 in situ hybridization in the CA3 region were similar to those described for the CA1 region, except that the intensity of the reaction in postpubertal females was less intense and some areas of the oriens and radiated layers were less positive than the rest. In prepubertal males, higher positivity was observed in CA3 than in CA1 and postpubertal males, and positivity in the pyramidal layer was predominant over the rest of the layers, as shown by the densitometric gradient.

**Figure 8:**
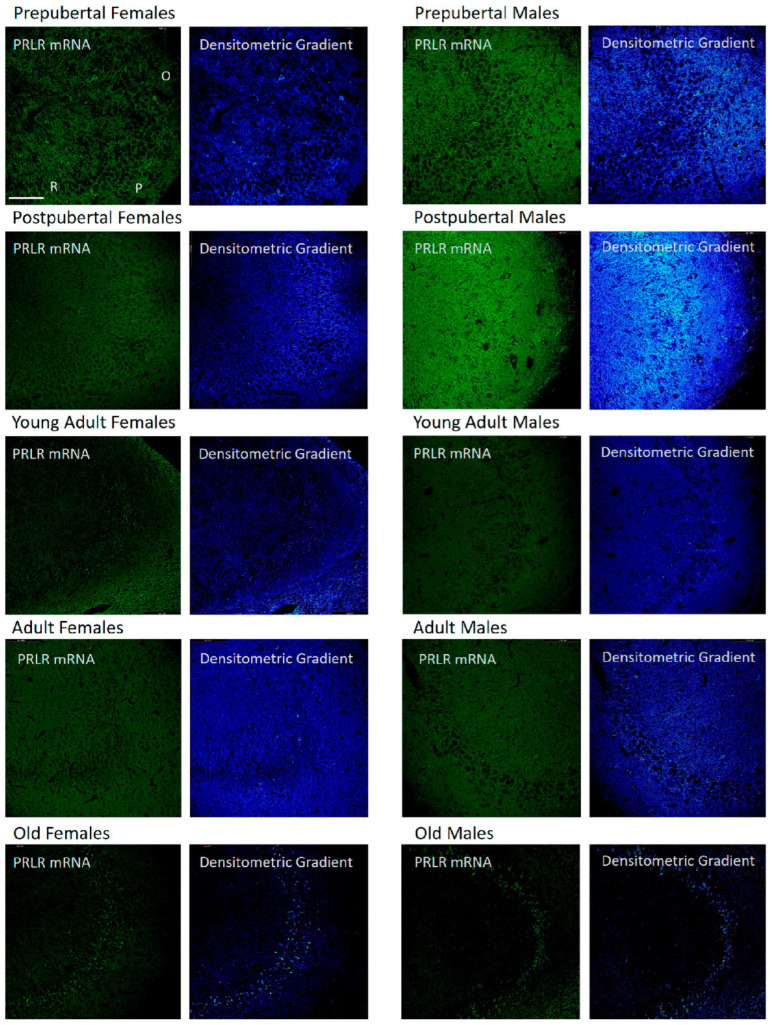
CA3 pyramidal neurons were the preferred site for localization of Box2 mRNA expression by in situ hybridization. In adult and old animals of both sexes, it was the only site of localization in most cases. The greatest expression was seen in postpubertal males, who presented a reaction in all CA3 layers, and the reaction was very evident in some secondary neurons of the stratum oriens. (O: Oriens layer, P: Pyramidal layer, R: Radiate layer.) Scale bar: 116 μm.

### 2.9. Distribution of Box2 mRNA in the Dentate Gyrus by In Situ Hybridization (Figure 9)

The results found in the dentate gyrus almost overlap with those described for CA3, now in the molecular, granular, and polymorphic layers. In postpubertal males, positivity was higher in the molecular layer.

In pre- and postpubertal females, Box2 mRNA-positive subgranular cells and secondary neurons of the polymorphous layer appeared. In the postpubertal stages, all subgranular cells were positive and some presented extensions towards the granular layer. In the rest of the age groups, this positivity disappeared, except in some subgranular cells isolated from old females. The findings regarding subgranular cells and secondary neurons in pre- and postpubertal males were the same as those in females. However, they were not observed in old males, and although weakly positive, subgranular cells appeared in young adult and adult males.

**Figure 9:**
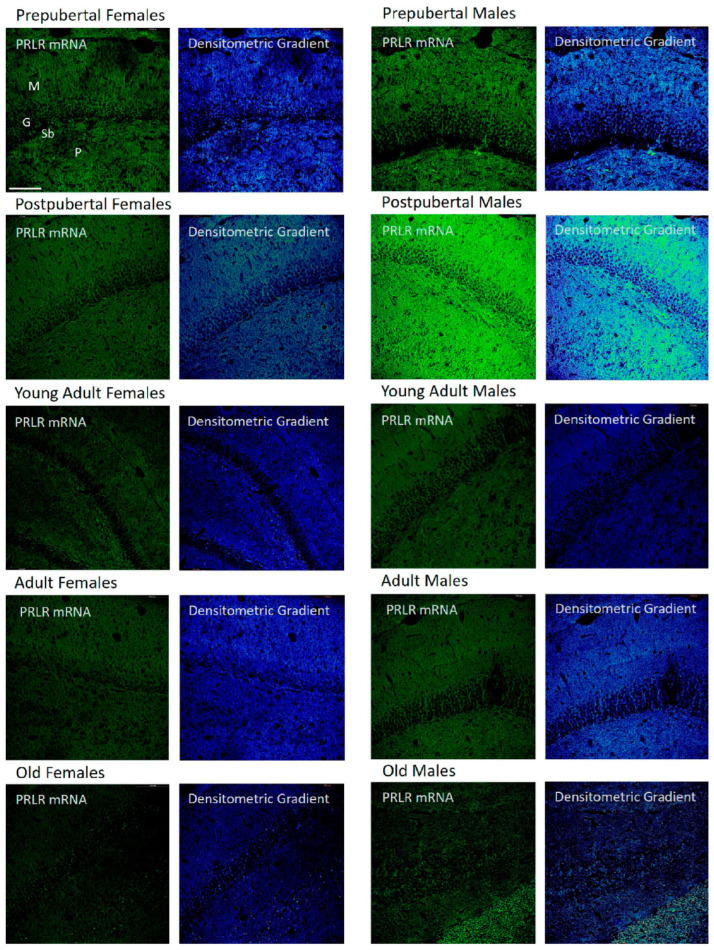
In situ hybridization showed that Box2 mRNA was well expressed in granular cells, except in young adults and adult females. In both sexes, this expression was more evident in the pre- and postpubertal stages than in the rest of the ages studied. The expression of Box2 mRNA was very strong in the three layers of the dentate gyrus of postpubertal males who also showed intensely positive subgranular cells. Scattered positive subgranular cells also appeared in prepubertal and postpubertal females. (M: Molecular layer, G: Granular layer, Sb: Subgranular layer, P: Polymorphic layer.) Scale bar: 116 μm.

## 3. Discussion

Prolactin is a hormone with a specific receptor and develops its biological roles acting on these receptors. The expression of the prolactin receptor in the brain has been widely described in the literature.

Radioautography, binding assays, immunohistochemistry, and in situ hybridization have provided evidence for the expression of the prolactin receptor throughout several brain regions, using radioautography [23,24], binding assays [25], immunohistochemistry [26,27,28], and in situ hybridization [29,30,31,32]. A description of the prolactin receptor expression in the brain using a novel transgenic reporter mouse strain has been reported [33]. These studies have demonstrated the widespread distribution of PRL receptors in the brain, including the medial periventricular hypothalamic nuclei, from the preoptic region through to the caudal arcuate nucleus, and in several extra-hypothalamic regions, such as the bed nucleus of the stria terminalis and the posterodorsal medial amygdala.

Prolactin receptor expression in the hippocampus has been demonstrated in in vivo and in vitro studies [21,22,27,34,35]. In 1996, it was reported that the expression of the receptor changes depending on the hormonal status of female rats, with them being at different stages of the estrous cycle, pregnancy, or lactation [36]. The expression of the isoforms differed between the short and long forms, with the short form being more stable and less sensitive to hormonal changes in the serum.

Even during pregnancy, the overexpression of the long isoform occurs earlier than that of the short isoform. These differences have been reported in the choroid plexus but not in the hypothalamic nuclei [37]. The short isoform seems to be the most common in the choroid plexus, whereas the long isoform is practically undetectable [19]. The overexpression of the prolactin receptor has been associated with maternal behavior, such as licking and nursing pups [37,38] and stressful situations [39]; however, other studies claim that maternal rats express fewer receptors than virgin diestrus rats [40].

Long and short isoforms of the prolactin receptor have been described in the hippocampus [34,39,41,42,43]; however, the long isoform is more highly expressed in the hippocampus and in certain types of cancer, which could be related to cell proliferation [39,44,45,46].

Even at low levels, prolactin receptor gene expression in the hippocampus has been confirmed by qPCR in rats [34,35], supporting the idea that prolactin plays a role in this brain region mediated by its receptor.

Our qPCR results support the published literature, indicating that the long form is more highly expressed than the short form is. Considering that the long form has a higher affinity for STAT5 phosphorylation, it could be hypothesized that this intracellular pathway is favored over the others.

However, there are no studies in the literature that have analyzed prolactin receptor expression in the hippocampus by qPCR and in situ hybridization. In this study, the presence of receptor mRNA was confirmed by qPCR and in situ hybridization, and it was localized in all regions of the hippocampus and dentate gyrus. However, our results show that there are variations in prolactin receptor expression depending on age and that, in most cases, this expression is sexually dimorphic.

The fact that we could detect the receptor in all age groups implies that prolactin plays a role in the hippocampus throughout life, and not exclusively in the mating, maternal, and perinatal periods. Although, the predominance in pre- and postpubertal animals and the differences related to age and sex for each isoform suggest that prolactin acting on its receptor plays a key role in the hippocampus and dentate gyrus in the early stages of life, mainly in prepubertal females and pre- and postpubertal males.

There is no clear and conclusive explanation for the peak expression of the prolactin receptor in postpubertal males and its distribution in somas, dendrites, and probably axons of the hippocampal neurons. The future analysis of the role played by prolactin in males immediately after puberty may explain the reason for this dimorphism. This may be related to the fact that in male rats, there is an increase in serum prolactin levels between puberty and adulthood [47,48].

More than 300 different biological functions have been described for prolactin, some of which are related to the central nervous system, including the stimulation of neurogenesis, modulation of stress responses, reduction in anxiety, transport of calcium, and regulation of the immune system [49,50,51]. In addition, this hormone has been implicated in hippocampal neuroprotection [4,6,7,35,39,46,52], with Ammon’s horn pyramidal neurons benefiting most from the protective role of prolactin against neuronal damage [3,4]. On the other hand, prolactin has been associated with neurogenesis in the subgranular zone of the dentate gyrus [4,5,39,53,54,55].

In our study, the expression of both isoforms of the prolactin receptor decreased in adult and old rats of both sexes, which is relevant to the decrease in Box2 expression in old females. It can be hypothesized that hippocampal aging in these animals may be related to a decrease in the PRL expression of the prolactin receptor, among other factors.

Most of the studies on neurogenesis and neuroprotection mentioned above have been carried out in young adult rodents, as well as the response to prolactin administration and the consequent activation of intracellular signaling pathways that have been described in certain circumstances such as brain development [55], in the brain’s response to injury [35,42,56,57,58], or in vitro studies of hypothalamic fragments or glial cells in culture [59,60,61]; it is unknown if these effects can occur in old rats. Sexual dimorphism in the hippocampus has been studied in young adult rats, where the administration of prolactin enhanced the synaptic plasticity by the activation of its receptor [62]. While memory-impaired young rats do not show the upregulation of prolactin or its receptor gene expression, old rats showed such upregulation, possibly acting as a neuroprotector [63]. As a result, an analysis of the response and possible increase in prolactin receptors in the hippocampus of old rats after the exogenous administration of prolactin could be an interesting route to understand brain aging.

## 4. Materials and Methods

### 4.1. Animals and Groups to Study

A total of 100 rats (*Rattus norvergicus*) were used in this study between 2019 and 2024. Fifty of these were used for histological analysis, and the other 50 were used for molecular analysis. Rats that developed pathologies due to aging, such as diabetes or tumors, particularly prolactinomas, were discarded at the time of sacrifice. The study groups were divided in the following manner: Prepubertal group: 4 weeks old, they were already separated from the mother and were not lactating rats; however, they had not yet reached puberty. Postpubertal group: 6 weeks old, where they reached puberty within 5 days of the day of sacrifice. Young adult: 4–5 months old, virgin, and not exposed to mating. Adult: 9–10 months old, virgin, and not exposed to mating. Old: 15+ months, virgin, not exposed to mating, and unable to reproduce anymore. Each of these groups had 10 animals of each sex; 5 were used for histological studies, and the other 5 for molecular studies. Every female rat (from postpubertal to adult age) was sacrificed during the estrous stage of the estrous cycle. All animals were kept under standard conditions for experimental rats, with a light/dark cycle of 12/12 h and free access to food and water resources.

All methods and procedures involved in this experiment were approved by the Ethics Committee of the University of Salamanca (registration number: 1146) and will be conducted in accordance with the animal care guidelines of the European Common Council (86/609/EEC) and Spanish regulations (Royal Decree 1201/2005), making significant efforts to minimize both suffering and the number of animals used.

### 4.2. Animal Sacrifice and Sample Collection

For histological studies, animals were sedated with equitesin (1 mL/250 g b.w., intraperitoneal), a mixed solution of pentobarbital (2.425 g, Sigma-Aldrich, St. Louis, MO, USA), chloral hydrate (10.625 g, ITW Reagents Panreac, Barcelona, Spain), ethanol (99.9% 24.125 mL), propylene glycol (103.25 mL), magnesium sulfate (5.25 g), and bi-distilled water (115.7 mL). After opening their thorax, they were killed via trans-cardiac perfusion by substituting the blood with heparinized saline serum (0.9% NaCl) and then perfused with paraformaldehyde (4%). After careful dissection, the brains were removed from the skull and kept in paraformaldehyde (4%) for the next 24 h. To preserve the tissues for freezing, the brains were submerged in increasing solutions of sucrose in phosphate saline buffer (PBS 0.1 M, 0.9% NaCl, pH 7.4) and then frozen, and serial sections of 20 µm thickness were obtained using a Microm^®^ cryostat (Microm Internaitonal GmbH, Wallford, Germany). Sections were introduced into wells with PBS and then fished to be placed on microscope slides.

**Figure 10:**
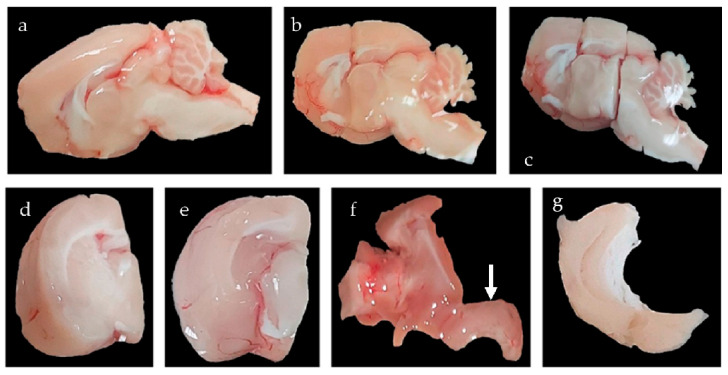
Isolation by microdissection of the hippocampus. (**a**): Medial vision of the rat brain after making a sagittal cut through the interhemispheric cleft. (**b**): Frontal cut at the intersection of the fornix column and the corpus callosum; (**c**): Frontal cut that crosses the cephalic portion of the superior colliculus; (**d**): View of the block obtained after the two frontal cuts; (**e**): Separation, below the corpus callosum, of the diencephalic elements and striatum, leaving the lateral ventricle and choroid plexuses visible; (**f**): Separation of the hippocampus (arrow in lower right) from the rest of the cerebral cortex; (**g**): Isolated hippocampus without the blood vessels and choroid plexuses.

For analytical and molecular studies, animals were sedated with equitesin, a mixed solution of pentobarbital, chloral hydrate, ethanol, propylene glycol, magnesium sulfate, and bi-distilled water. After careful dissection, brains were removed from their skull and kept in synthetic cerebrospinal fluid for dissection of the hippocampus (gasified aqueous solution of 3 mM KCl, 1.25 mM NaH_2_PO_4_, 28 mM NaHCO_3_, 14.56 g sucrose, 0.36 g Dextrose, 2 mL 1 mM MgCl_2_, 100 mL of 1 M CaCl_2_, and 1 mL mL 0.6 M sodium pyruvate) using a protocol designed specifically for this study. To obtain the cerebral block containing the hippocampus, according to Patxino’s Rat Brain stereotaxic atlas [64], two frontal cuts were made: one cephalic cut at the intersection of the fornix column with the corpus callosum and one caudal cut in the cephalic part of the superior colliculus. In the third step, dissecting below the corpus callosum towards the lateral ventricle, the striatum and thalamus were moved medially. Finally, through careful dissection, the hippocampus was separated from the rest of the cortex following its dorsolateral limit, which was provided by the corpus callosum, until it was completely separated (Figure 10). Once isolated, the hippocampus was frozen by immersion in liquid nitrogen and stored in a freezer at −80 °C.

### 4.3. In Situ Hybridization

After drying, the frozen slides were submerged in an acetylated buffer for 10 min. Subsequently, they were washed with distilled water and dried in a stove at 37 °C for 20 min. Pre-hybridization was performed using Omnibuffer (Wak-Chemie Medical GmbH, Steinbach (Taunus), Germany in a HybAid Omnislide thermocycler (Thermo Fisher Scientific, Waltham, MA, USA) in a humidity chamber for 1 h at 37 °C. Hybridization was performed in the same chamber at 37 °C overnight with the following 5′-biotinylated probes:

Sense probes: Rat PRLR_S1 (short isoform): {Btn} TCTTGGGTAAAACTCTCCGAGGATAAAC,

Rat PRLR_L1 (long isoform): {Btn} AGAAACCTTACCCTACCGTAATCGGCA.

Antisense probes: Rat PRLR_S1: {Btn}AGAACCCATTTTGAGAGGCTCCTATTTG,

Rat PRLR_L1: {Btn}TCTTTGGAATGGGATGGCATTAGCCGCT.

After hybridization, the slides were submerged twice in HybAid Omnislide washing module (Thermo Fisher Scientific, MA, USA) for 5 min in astringent washes with 0.1% sodium citrate buffer (SSC buffer) at 37 °C. The samples were washed once with the same buffer at room temperature for 5 min. To avoid false-positive detection, after hybridization and astringent washes, the samples were treated with RNase.

Biotin was detected by immunohistochemistry. The samples were washed twice in TBS for 5 min at room temperature. Blocking was performed with BSA for 30 min and then incubated overnight at 4 °C in a humidity chamber with primary mouse antibody anti-biotin (DAKO. M0743) at a dilution of 1/350 in TBS.

After washing with TBS twice for 5 min at room temperature, the samples were incubated for 3 h at room temperature with Goat Anti-Mouse antibody conjugated with Alexa Fluor 488 (Abcam, Cambridge, UK. Ab150113) which was diluted to 1/800 in TBS. Cells were washed twice with TBS and Hoechst 33342 (Cayman Chemical, Ann Arbor, MI, USA. 15547) at a dilution of 1/1500 and were incubated for 15 min. Finally, after washing with TBS twice and leaving the slides for another 5 min in distilled water, Fluoromont (Sigma-Aldrich, St. Louis, MO, USA. F4680) was applied, and the cover was placed and sealed.

**Figure 11:**
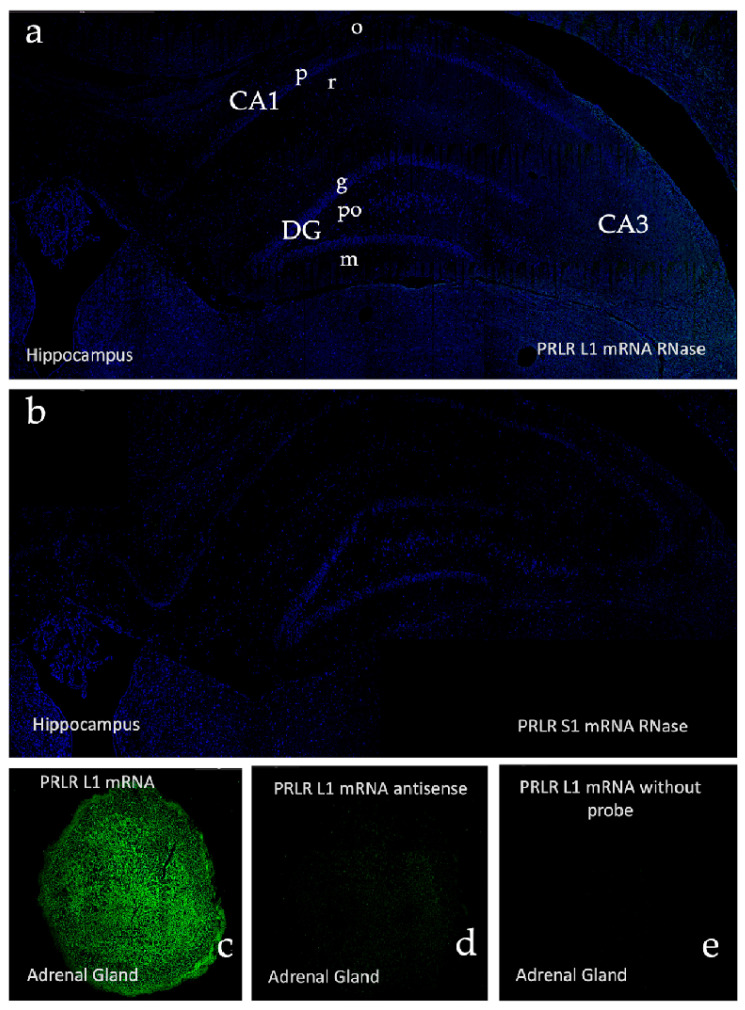
Microphotographs showing the absence of a signal in the control tests for in situ hybridization of Box1 (RPRL S1 mRNA) and Box2 (RPRL L1 mRNA) (**a**,**b**) hippocampus after previous treatment with RNase, (**c**) the presence of a signal in a positive control performed in the adrenal gland, (**d**) adrenal gland after previous incubation with antisense probe, and (**e**) adrenal gland in the absence of probe. (o: stratum oriens; p: stratum pyramidale; r: stratum radiatum; CA1-3: Cornu Ammonis 1-3; g: granular layer, po: polymorphic layer; m: molecular layer).

Hybridization controls were performed on adrenal and hippocampal sections: positive control for rat PRLR-L1 and RPRL-S1—hippocampus and adrenal gland, obtained from untreated female rats in the estrous cycle. Negative control: same quantity of sense and antisense probes. Negative control: pretreatment of tissue with RNase enzyme. Negative control: development of a reaction in the absence of probe hippocampus and adrenal gland, obtained from untreated female rats in the estrous cycle. Figure 11 shows some of the results obtained for the positive and negative controls for PRLR-L1 (Box2) and PRLR-S1 (Box1) in situ hybridization in the hippocampus and adrenal gland.

### 4.4. Image Analysis

Images were obtained using a confocal microscope Stellaris (Leica Microsystems GmbH, Wetzlar, Germany). Magnification was ×20 and the resolution was 2048 × 2048. The CA1, CA3, and DG hippocampal regions were photographed. A Z-stack was performed and exported images were the maximum exposure composition of all the photos made of the Z-axis in the same X and Y positions. The same pinhole, gain, brightness, and contrast were used to obtain digital microphotographs.

### 4.5. Immunofluorescence Gradient Python Script

Immunofluorescence images were processed using a custom Python script to generate intensity gradient images and determine the densitometric values for every pixel. The script assigns intensity values that range from 0 to 255 in the original image and replaces that pixel on a scale where cold colors represent low intensity values and warm colors represent higher values, resulting in an image where the most intense pixels can be easily detected. The main difference between this gradient and others used in the industry is that the purple color has been removed from the scale, as it usually appears between blue and teal colors, and we consider purple a warm color, confusing the interpretation of the images. The script was programmed in Python 3.89.6 by Julian Happel [65]. IF-Grad: Fluorescent image gradient converter (v 0.1.2)-free computer software https://github.com/chaotix1992/IF-Grad (accessed on 17 November 2023).

### 4.6. q-PCR Technique

#### 4.6.1. Messenger RNA Extraction and Complementary DNA Synthesis

After hippocampal dissection, the tissue was immediately frozen in liquid nitrogen and stored at −80 °C until RNA isolation. Isolation was performed using RNeasy Mini and QIAshredder Kits (Qiagen N.V., Venlo, The Netherlands #74104 and #79654) according to the manufacturer’s instructions. RNA was stored at −80 °C and used for cDNA synthesis using random primers and MultiScribe Reverse Transcriptase (Thermo Fisher Scientific). High-capacity RNA-to-cDNA^TM^ kit #4387406, with the following mix per sample: 10 μL of 2× buffer mix, 1 μL 20× enzyme, up to 2 μg or up to 9 μL of RNA sample, and nuclease-free water to a total volume of 20 μL, in a thermal cycler with the following program settings: 10 min at 25 °C, 120 min at 37 °C, and 5 min at 85 °C. The cDNA obtained was stored at −20 °C till qPCR.

#### 4.6.2. Cycling Chain Reaction

Real-time quantitative PCR (q-PCR) was performed using gene-specific primers spanning exon–exon junctions in the target cDNA, AmpliTaq Gold DNA polymerase, and SYBR Green I detection kit in a QuantStudio™ 3 System (Thermo Fisher Scientific). For a total volume of 20 µL per well, 2 µL of cDNA previously diluted to 20 ng/μL, 10 µL of SYBR™ Select Master Mix (Thermo Fisher Scientific 4472908), 0.1 µL of forward primer previously diluted at 20 µM, 0.1 µL of reverse primer previously diluted at 20 µM, and 7.8 µL of double-distilled water were mixed.

Holding was carried out in two steps at 50 °C and 95 °C for 2 min and 10 min, respectively. Forty PCR cycles at 95 °C for 15 s and 60 °C for 1 min. The melting curve was carried out by 3 steps: 1.6 °C/s at 95 °C for 15 s, 1.6 °C/s at 60 °C for 1 min, and 0.15 °C/s at 95 °C for 5 s. The mRNA abundance of the target genes in each sample was normalized based on *Hprt-1* expression.

#### 4.6.3. Primers and Data Normalization

Primers were tested to determine whether they were optimal for our experiments. Primers for prolactin receptor were tested in the adrenal gland and hippocampus. The ratio was calculated using Hprt1 as the housekeeping gene (HKG), whose expression was stable in all samples, using the following equation:

Ratio (%) = 2^(−ΔCt)^ × 100, where ΔCt = Average target Ct − Average HKG Ct

*Primer sequences 5′-3′:* Prolactin receptor Box1 forward AAAGTATCTTGTCCAGACTCGCTG,           Prolactin receptor Box1 reverse AGCAGTTCTTCAGACTTGCCCTT,          Prolactin receptor Box2 forward GCAGGTGAATGTTTCCTTGTC,         Prolactin receptor Box2 reverse CTTGCTTTCGTCCTACTTGTTC,  *Hprt-1* forward CCCAGCGTCGTGATTAGCGAT,        *Hprt-1* reverse CGAGCAAGTCTTTCAGTCCTGTCCATA.

### 4.7. Statistic Treatment of Data

The values obtained were statistically analyzed using GraphPad Prims (8.4.3 version). Descriptive analysis (where minimum, maximum, range, arithmetical mean, standard deviation, and standard error of the mean were calculated) and two-way analysis of variance (ANOVA) were performed. The significance of multiple comparisons was checked using Tukey’s test. The Confidence Interval was 95%, where the *p*-value was significant if *p* < 0.05, and very significant if *p* < 0.01 or more.

## Data Availability

The data presented in this study are available upon reasonable request from the corresponding author.

## Abbreviations

The following abbreviations are used in this manuscript:

Akt	Protein Kinase B, also known as Akt, is the collective name for a set of three serine/threonine-specific protein kinases. Oncogene isolated from tumors produced by the murine retrovirus AKT8
Btn	Biotinylated
CA1	Hippocampal Cornus Amonii 1 region
CA3	Hippocampal Cornus Amonii 3 region
cDNA	Complementary deoxyribonucleic acid
DG	Dentate Gyrus
Hprt1	House-keeping genes. It is a gene that provides instructions for producing an enzyme called hypoxanthine phosphoribosyltransferase 1
JAK2	Janus Kinase 2
MAPK	mitogen-activated protein kinase
MEK	Also known as MAPKK, it is a kinase that phosphorylates and activates MAPK (MAPK kinase/ERK kinase)
mRNA	Messenger ribonucleic acid
mTor	Mammalian target of rapamycin
PBS	Phosphate saline buffer
PI3K	phosphatidyl-inositol 3-kinase
PRLR	Prolactin receptor
qPCR	Quantitative polimerase chain reaction
Raf	Ras-activated factor; it is a kinase that phosphorylates and activates MEK
Ras	Retrovirus-associated sequences of Monomeric G protein; it is a G protein, involved in the MAPK activation pathway by growth factors and mitogens
Src	Steroid receptor coactivator
SSC	Sodium citrate solution
STAT3	Signal transducer and activator of transcription 3
STAT5	Signal transducer and activator of transcription 5
TBS	Trizma saline buffer
